# Supplementary figures and images for: The impact of size on middle-ear sound transmission in elephants, the largest terrestrial mammal
Source: PLoS One. 2024 Apr 10;19(4):e0298535. doi: 10.1371/journal.pone.0298535 (PMC11006165; doi:10.1371/journal.pone.0298535)

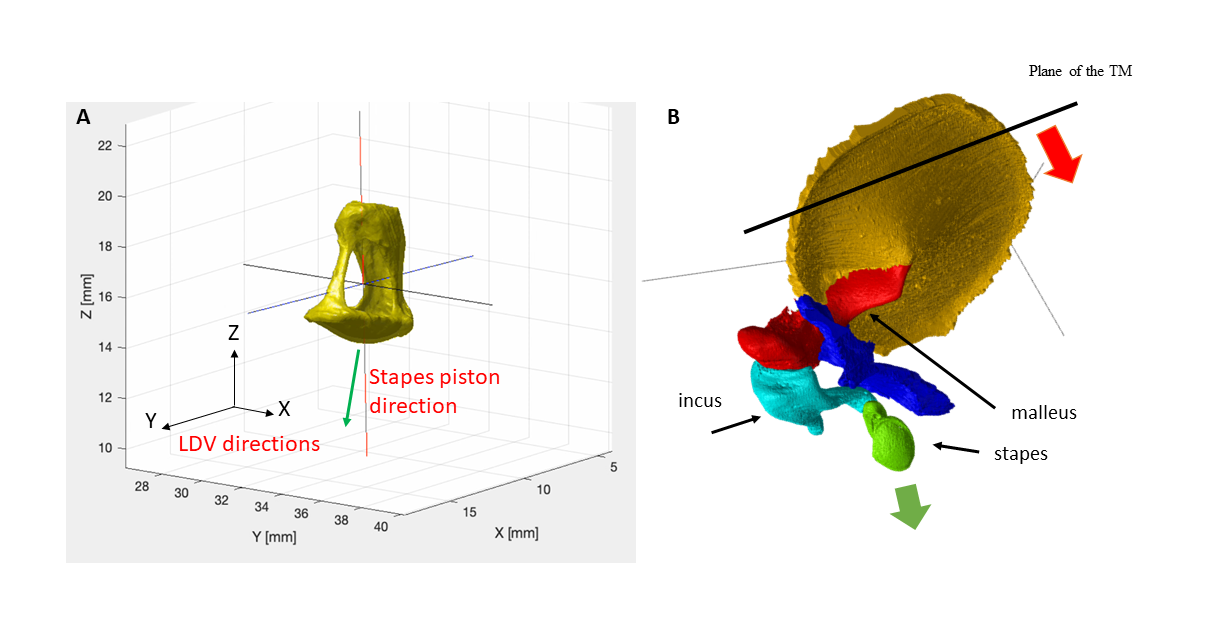

Supplement: S1 Fig — (A) Stapes piston direction (green arrow) calculate from the 3D LDV reference measurement and angle measurements. The measurement was made at the stapes head. (B) The measured 3D umbo velocity was projected to an umbo 1D velocity (red arrow) along a vector normal to the plane of the TM (the black line lies on this plane). The stapes velocity along its piston direction is shown by the green arrow. μCT reconstructions are for ETB2. (TIF) [file pone.0298535.s001.tif]

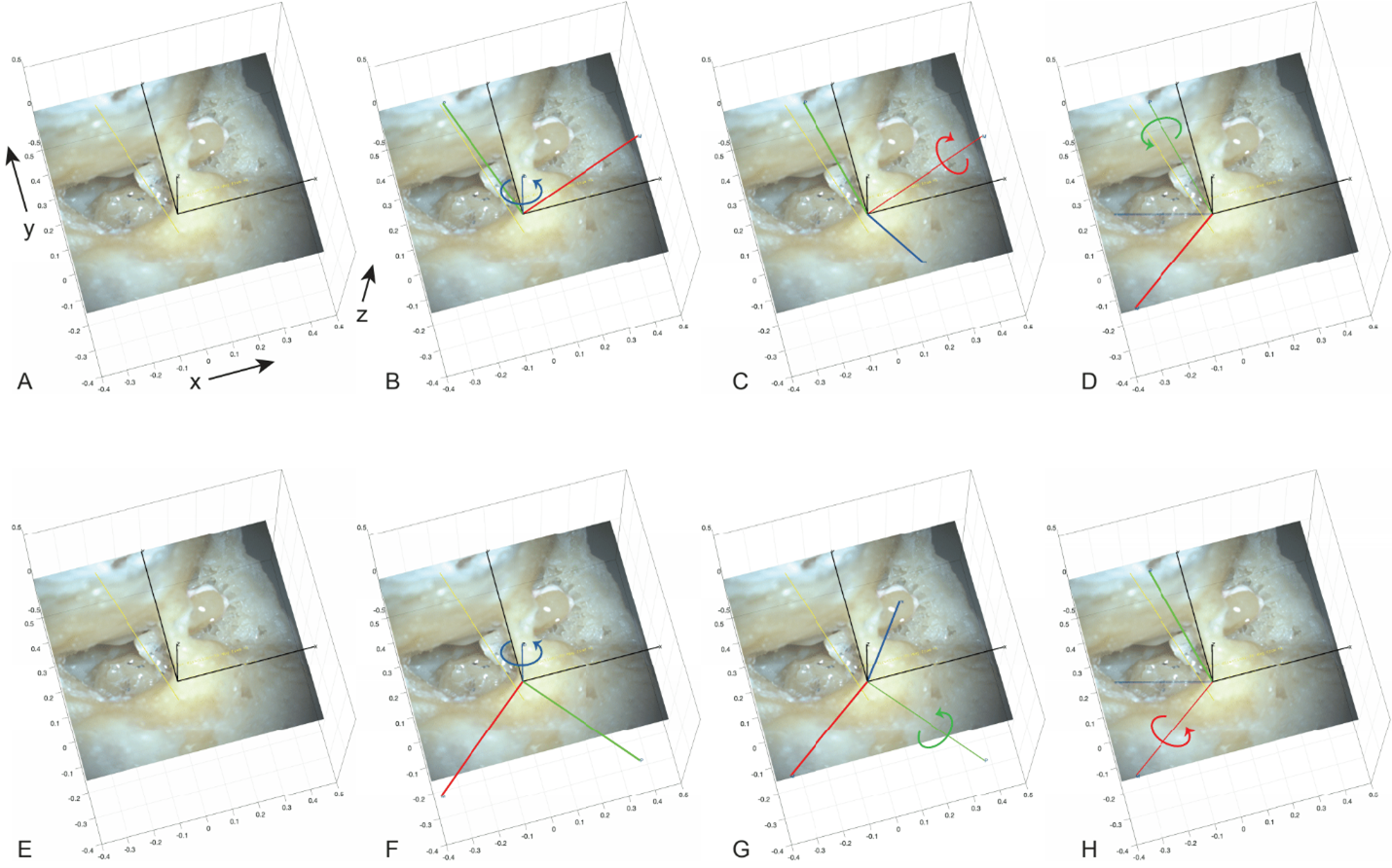

Supplement: S2 Fig — All panels show the extrinsic (x-y-z) reference frame in black, superposed over a photograph of the stapes and surroundings taken by the camera through the dichroic mirror on the 3D LDV and rotated slightly to show the z axis more clearly. The origin is at the center of the stapes footplate. (A-D) Rotations in TB20 in the order they were measured; (E-H) Rotations in the order used by the Spatial Math Toolbox. (A) Extrinsic reference frame; (B) rotation of +20° about the z axis (blue arrow) to align the y axis (green) to the projection of the stapes piston direction in the x-y plane–the rotated x and y axes are shown in red and green respectively; (C) additional rotation of +40° about the (new) x axis (red arrow) = elevation from the x-y plane–the rotated z axis is shown in blue; (D) additional rotation of –150° about the (new) y axis (green arrow) to arrive at the Maj-pist-min intrinsic reference frame (red-green-blue). (E) The extrinsic coordinate system as in (A); (F) rotation of –139° about the z axis (blue arrow); (G) additional rotation of –22° about the (new) y axis (green arrow); (H) additional rotation of +135° about the (new) x axis (red arrow). Panels (D) and (H) are identical, which shows that the two rotation sequences are equivalent. (TIF) [file pone.0298535.s002.tif]
