# Supplementary material for: The impact of size on middle-ear sound transmission in elephants, the largest terrestrial mammal
Source: PLoS One. 2024 Apr 10;19(4):e0298535. doi: 10.1371/journal.pone.0298535 (PMC11006165; doi:10.1371/journal.pone.0298535)
Supplement: S1 Table — Two orientations were used for ETB2: A with the tympanum proper intact (more posterior), and B with the tympanum proper removed (more anterior). Separate projections were applied to stapes footplate and incus velocities (SFP) and umbo velocities (UMB), as the stapes and umbo piston velocities were in slightly different directions. ETB7 Rz is the same as Azimuth. (DOCX) [file pone.0298535.s003.docx]

**Supporting text for: “The impact of size on middle-ear sound transmission in elephants, the largest terrestrial mammal”**

Authors: Caitlin E. O’Connell-Rodwell, Jodie L. Berezin, Anbuselvan Dharmarajan, Michael E. Ravicz, Yihan Hu, Xiying Guan, Kevin N. O’Connor, Sunil Puria

| **Specimen** | **Piston Direction** | **Azimuth** | **Elevation** | **Rotation** | **Rz** | **Ry** | **Rx** |
| --- | --- | --- | --- | --- | --- | --- | --- |
| ETB2A | SFP | 95 | 5 | -170 | -84.1 | -9.96 | 174.9 |
| ETB2A | UMB | 95 | 25 | 0 | 95.2 | 0 | 25 |
| ETB2B | SFP | 40 | 40 | 105 | 152.6 | 47.7 | 107.1 |
| ETB2B | UMB | 27 | 0 | 0 | 27 | 0 | 0 |
| ETB3 | SFP | -75 | 0 | 0 | -75 | 0 | 0 |
| ETB3 | UMB | -45 | -15 | 0 | -45.5 | 0 | -15 |
| ETB6 | SFP | -100 | -5 | 0 | -100 | 0 | -5 |
| ETB6 | UMB | -80 | 0 | 0 | -78 | 0 | 0 |
| ETB7 | SFP | -20 | ~-5 | ~5 | -20.7 | 4 | -4 |
| ETB7 | UMB | -18 | -10 | 0 | -18 | 0 | -10 |
| TB18 | SFP | -20 | 70 | -70 | -88.8 | -18.7 | 82.9 |
| TB18 | UMB | 0 | -20 | 0 | 0 | 0 | -20 |
| TB19 | SFP | 20 | 20 | -90 | -70 | -70 | 90 |
| TB19 | UMB | 15 | 0 | 0 | 15 | 0 | 0 |
| TB20 | SFP | 20 | 40 | -150 | -139.6 | -22.5 | 135.9 |
| TB20 | UMB | 0 | 0 | 0 | 0 | 0 | 0 |

**S1 Table. Projection rotation angles (in degrees) for each temporal bone, as measured (Azimuth, Elevation, Rotation) and as recomputed for implementation in Z-Y-X order.** Two orientations were used for ETB2: A with the tympanum proper intact (more posterior), and B with the tympanum proper removed (more anterior). Separate projections were applied to stapes footplate and incus velocities (SFP) and umbo velocities (UMB), as the stapes and umbo piston velocities were in slightly different directions. ETB7 Rz is the same as Azimuth.
