# Supplementary material for: The impact of size on middle-ear sound transmission in elephants, the largest terrestrial mammal
Source: PLoS One. 2024 Apr 10;19(4):e0298535. doi: 10.1371/journal.pone.0298535 (PMC11006165; doi:10.1371/journal.pone.0298535)
Supplement: Attachment — Submitted filename: OCRetal_Ele_AC_PLOSONE_RebuttalLetter.docx [file pone.0298535.s011.docx]

Dear Dr. Andre van Wijnen and reviewers,

1/8/2024

We are pleased to submit the revisions for our research article “The impact of size on middle-ear sound transmission in elephants, the largest terrestrial mammal” (manuscript ID: PONE-D-23-31155). We have uploaded one version of the revised manuscript with tracked changes and one without to the submission portal. We thank the reviewers for their helpful comments on the manuscript. Below, we provide responses to the editor and the reviewers.

Editor comments:

1. Formatting in PLOS ONE’s style requirements: We have made all style and formatting changes necessary to fulfill PLOS ONE’s style requirements. Please note that we have moved the methods from the end of the manuscript to after the introduction section.
2. Participant consent and ethics statement: We have included the appropriate participant consent and ethics statement at the beginning of our methods section. Our statement reads as follows: “This study was performed in line with the principles of the Declaration of Helsinki. Human post-mortem specimens: Human-subject research in this manuscript meets the criteria for exemption from the Mass General Brigham IRB requirements in DHHS regulations (45 CFR 46), which would include participant consent. Specifically, the research falls under Exemption 4: Research involving the collection or study of existing data, documents, records, pathological specimens, or diagnostic specimens, if these sources are publicly available or if the information is recorded by the investigator in such a manner that subjects cannot be identified directly or through identifiers linked to the subjects. Human temporal bones were harvested from the Massachusetts General Hospital morgue in accordance with the appropriate Mass Eye & Ear Otopathology Laboratory protocols. Elephant post-mortem specimens: No approval of research ethics committees were required to accomplish the goals of this study, because experimental work was conducted on post-mortem temporal bones donated from zoos and sanctuaries.”
3. Acknowledgements section: Per your request, we have removed funding-related text from the acknowledgements section.
4. Funding statement: We have removed all funding information from the manuscript. Our funding statement is correct and does not need to be amended.
5. Ethics statement: As stated above, we have updated the methods section to include a statement regarding waived consent from the ethics committee.
6. Supporting information file S1_Data: We have revised and reuploaded this zipped file.

Reviewer comments (black bullet points are reviewer comments, open circles are author responses):

Note: black bullet points are reviewer comments, open circles are author responses. Page and line numbers are for the tracked changes version of the manuscript.

Reviewer #1:

- Line 77: “whereas it was reported as 9.6x larger (454 vs. 68.3 mm2)”. It should be “6.6x” larger.
  - Thank you, we have adjusted this in the text and double checked all other calculations in the manuscript to ensure accuracy.
- In Table 1, the TM surface areas between African and Asian Elephants are significantly different (606.3 vs. 403.6), suggesting species differences. Indeed the TM surface area of Asian Elephant is not that much different from Nummela et al.
  - Thank you for this observation, the difference in TM surface area between the Asian and African elephant is significant for both our study and Nummela [1]. We have included a statement in the first paragraph of the results section that acknowledges this (page 13-14, lines 294-298).
- How was the thickness of the TM quantified? Given the thickness of the human TM quantified in this study (223 um) is more than twice of the reported human TM thickness (80~100 um), please give more details and justify the results.
  - TM thickness was calculated using the “wall thickness” tool in Synopsys Simpleware (page 10, line 215) which calculates the average thickness across the entire TM. Calculating the thickness estimates of the TM are difficult using µCT due to the low x-ray absorption of soft tissues [2]. Because of this, our thickness estimates were approximately double to triple the average value of TM estimates from OCT scans for humans. The thickness of the human TM has been reported to have a wide range (from 30–150 µm), with many researchers adopting an average human TM thickness of 74-100 µm (discussed in [3]). Therefore, we assume our thickness, volume, and mass calculations are smaller than what is expected, but the relative size difference between elephant and human should be representative.
  - As such, we have amended the results section (page 14, lines 299-304) and table 1 caption (page 15-16, lines 330-337).
- Is the density of elephant middle ear same as the density of human ear when calculating the mass?
  - Nummela [1] reported to have measured the densities of some human, seal, and elephant ossicles with density values between 2.0 and 2.3. Since the specifics were not listed for elephant, we assumed the densities were similar enough to human, and used the human density values presented in Sim & Puria [4] for elephant estimates. We have added a statement in the “micro-CT imaging and segmentation” methods section to clarify this (page 10, lines 220-223).
- In Table 3, some mean values do not seem to match with individual values listed there. For instance, in Human HF column, how could 25 and 24 yield mean of 9.5? Please double check all your calculations of means and SDs and revise text accordingly.
  - We have double checked this table and confirmed all values are correct. We stated in the table caption that we subtracted 15 µs from human mean estimates to account for the 5 mm ear canal length. To make this more obvious for readers, we have included an asterisk to draw attention to this note in the table caption.
- Given only a limited number of elephant ears were measured in this study, would it make more sense to look at individual ear rather than looking at the mean and SD? For example, in Table 3, in Elephant HF column, the umbo group delay from four elephant ears are 5, -2, 170, 62, which makes no sense to average them. Plots like those shown in S2_Fig are more meaningful.
  - While we still think it is valuable to show the overall trends for the human versus elephant velocity measurements, we agree that it would be helpful to see the variation between specimens. We’ve added S2_Fig into the main text as figure 4, presented right after the overall trends (page 18, lines 380-81; caption on page 19, lines 400-408).

Reviewer #2:

- Line 384: “Fig. 2H” instead of “Fig. 3H”
  - Thank you. After moving the methods, Fig 3H was now the correct figure to reference so it was left as is.
- Can you transfer your findings to the real life of elephants and speculate on the evolutionary benefits of their hearing?
  - Elephants communicate using low frequency vocalizations that propagate long distances, making it possible to maintain contact with extended family members [5, 6], share information about the environment and potential danger [7-9], as well as advertising reproductive states [5, 10, 11]. The ability to hear these low frequency sounds enables the elephant to maintain complex social relationships across many different landscapes.
  - The advantages of being able to communicate over long distances would have benefited elephants over evolutionary time. For the genus Loxodonta, as the climate became drier in Sub-Saharan Africa and the forest landscape shifted to a more open savannah, the ability to detect low-frequency sounds would have allowed individuals to remain in contact with family members while still being able to feed across longer distances. Since higher frequencies attenuate in dense forest habitats, where the genus Elephas evolved, low frequency vocalizations may have been selected for to facilitate communication in both the near and far field. As such, Asian elephants have fewer higher harmonics within in their low-frequency vocalizations than African elephants do [12]. The more robust middle ear of the Asian elephant could speak to different evolutionary pressures on the Elephas genus before, during or after their coexistence with Loxodonta in sub-Saharan Africa during the Pliocene. These differences might impact the hearing physiology between the species but there is still much we don’t know about elephant communication and evolutionary pressures on ancestral species, limiting our ability to make any conclusions.
  - We have included this response in the last section of the discussion section in the manuscript (page 34).
- Are the findings of the two species (African and Asian elephant) comparable? Are the differences species or more age related?
  - Because of our small sample sizes, we were not able to make any conclusions about species or age-related differences between African and Asian elephant middle ears. Our only African elephant specimen was a four-month-old calf, and two of our Asian elephant specimens were calves and one was 72 years old, making it difficult to determine any meaningful differences. For example, we did not see any obvious differences between the adult Asian elephant (ETB6) and the calves (ETB3, ETB7), nor differences between the Asian elephant calves and African elephant calf (previously S2_Fig, now Fig 4). However, as Reviewer #1 points out, the difference in the TM surface area between African and Asian elephants is large. In our results, the weight of the adult Asian elephant ossicular chain was about 1.5x heavier than the infant African elephant, while Nummela found that the ossicle masses were 1.2x heavier for the Asian elephant than African, suggesting there might be an impact of age on the weight of the ossicles. Overall, based on our findings and Nummela’s, the Asian elephants appear to have smaller TM surface areas and heavier ossicles than African elephants, though the sample sizes for both studies were small.
  - The differences between the species might be due to their different ecologies, as described in our response to the previous question.

Warm regards,

Dr. Caitlin O’Connell-Rodwell, Ph.D.

Instructor

References

1.	Nummela S. Scaling of the mammalian middle ear. Hearing Research. 1995;85:18-30. doi: <https://doi.org/10.1016/0378-5955(95)00030-8>.

2.	Buytaert JA, Salih WH, Dierick M, Jacobs P, Dirckx JJ. Realistic 3D computer model of the gerbil middle ear, featuring accurate morphology of bone and soft tissue structures. J Assoc Res Otolaryngol. 2011;12(6):681-96. Epub 2011/07/14. doi: <https://doi.org/10.1007/s10162-011-0281-4>. PubMed PMID: 21751073; PubMed Central PMCID: PMCPMC3214243.

3.	van der Jeught S, Dirckx JJ, Aerts JR, Bradu A, Podoleanu AG, Buytaert JA. Full-field thickness distribution of human tympanic membrane obtained with optical coherence tomography. J Assoc Res Otolaryngol. 2013;14(4):483-94. Epub 2013/05/16. doi: <https://doi.org/10.1007/s10162-013-0394-z>. PubMed PMID: 23673509; PubMed Central PMCID: PMCPMC3705083.

4.	Sim JH, Puria S. Soft tissue morphometry of the malleus-incus complex from micro-CT imaging. J Assoc Res Otolaryngol. 2008;9(1):5-21. Epub 2008/03/04. doi: <https://doi.org/10.1007/s10162-007-0103-x>. PubMed PMID: 18311579; PubMed Central PMCID: PMCPMC2536804.

5.	Poole JH, Payne K, Langbauer Jr. WR, Moss CJ. The social contexts of some very low frequency calls of African elephants. Behavioral Ecology and Sociobiology. 1988;22:385-92.

6.	McComb K, Reby D, Baker L, Moss C, Sayialel S. Long-distance communication of acoustic cues to social identity in African elephants. Animal Behaviour. 2003;65(2):317-29. doi: <https://doi.org/10.1006/anbe.2003.2047>.

7.	Soltis J, King LE, Douglas-Hamilton I, Vollrath F, Savage A. African elephant alarm calls distinguish between threats from humans and bees. PLoS One. 2014;9(2):e89403. Epub 2014/03/04. doi: <https://doi.org/10.1371/journal.pone.0089403>. PubMed PMID: 24586753; PubMed Central PMCID: PMCPMC3935886 Animal Kingdom Cast Conservation Program supported the project. Joseph Soltis and Anne Savage are employed by Disney's Animal Kingdom. There are no patents, products in development or marketed products to declare. This does not alter the authors' adherence to all the PLOS ONE policies on sharing data and materials, as detailed online in the guide for authors.

8.	Sharma N, Prakash S V, Kohshima S, Sukumar R. Asian elephants modulate their vocalizations when disturbed. Animal Behaviour. 2020;160:99-111. doi: <https://doi.org/10.1016/j.anbehav.2019.12.004>.

9.	Nair S, Balakrishnan R, Seelamantula CS, Sukumar R. Vocalizations of wild Asian elephants (Elephas maximus): structural classification and social context. J Acoust Soc Am. 2009;126(5):2768-78. Epub 2009/11/10. doi: <https://doi.org/10.1121/1.3224717>. PubMed PMID: 19894852.

10.	Poole JH. Rutting Behavior in African Elephants: The Phenomenon of Musth. Behaviour. 1987;102(3/4):283-316.

11.	Langbauer WR, Payne KB, Charif RA, Rapaport L, Osborn F. African Elephants Respond to Distant Playbacks of Low-Frequency Conspecific Calls. Journal of Experimental Biology. 1991;157(1):35-46. doi: <https://doi.org/10.1242/jeb.157.1.35>.

12.	O'Connell-Rodwell CE, Hart LA, Arnason BT. Exploring the Potential Use of Seismic Waves as a Communication Channel by Elephants and Other Large Mammals. American Zoologist. 2001;41(5):1157-70. doi: <https://doi.org/10.1093/icb/41.5.1157>.
