# Supplementary material for: The impact of size on middle-ear sound transmission in elephants, the largest terrestrial mammal
Source: PLoS One. 2024 Apr 10;19(4):e0298535. doi: 10.1371/journal.pone.0298535 (PMC11006165; doi:10.1371/journal.pone.0298535)
Supplement: Attachment — Submitted filename: OCRetal_Ele_AC_PLOSONE_RebuttalLetter_RevisionsRound2.docx [file pone.0298535.s012.docx]

Dear Dr. Andre van Wijnen and reviewers,

1/19/2024

We are pleased to submit the minor revisions for our research article “The impact of size on middle-ear sound transmission in elephants, the largest terrestrial mammal” (manuscript ID: PONE-D-23-31155). We provide responses to the editor and the reviewers below.

Editor/journal comments:

- The reference list has been reviewed and we confirm it is complete and correct.
- We have made additional minor further clarifications regarding the TM calculations and densities on pages 10 and 15, as well as in the evolution section on pages 34-35, as per reviewer suggestions.

Reviewer comments:

Note: black bullet points are reviewer comments, open circles are author responses.

Reviewer #1:

- I appreciate the efforts the authors have made into their revisions. I only have a few minor suggestions.
  - Thank you!
- Line 216-217: “Nummela [2] reported human, seal, and elephant ossicle densities between 2.0 and 2.3.” Please add the unit of density after the numbers.
  - We have added the unit of density (mg/mm^3^).
- Table 3: Thanks for clarifying that you subtracted 15us to calculate human means. Looks like you also subtracted different times (varying from specimen to specimen) when calculating elephant means. However, are the values listed in Table 3 in the elephant columns from “after subtraction”? While the values listed in Table 3 in the human columns are from “before subtraction”? This is confusing, and I suggest you also list the values for the humans after subtraction.
  - You’re right, this was unnecessarily confusing. We have adjusted the table so that all values are “after subtraction” and have slightly modified the table caption with these changes.

Reviewer #2:

- The authors responded to all the reviewer's comments. I do not have any further comment and I would like to congratulate the authors for their excellent work.
  - Thank you!

Warm regards,

Dr. Caitlin O’Connell-Rodwell, Ph.D.

Instructor
